# Effects of Hydrogen Peroxide-Induced Oxidative Stress on Intestinal Morphology, Redox Status, and Related Molecules in Squabs

**DOI:** 10.3390/ani13040749

**Published:** 2023-02-19

**Authors:** Yajing Zhong, Tingting Ma, Zhiqi Fu, Ailing Chen, Jiahao Yu, Yanhua Huang, Jing Fu

**Affiliations:** 1 Innovative Institute of Animal Healthy Breeding, Zhongkai University of Agriculture and Engineering, Guangzhou 510225, China; 2 College of Animal Science and Technology, Zhongkai University of Agriculture and Engineering, Guangzhou 510225, China; 3 Guangdong Laboratory for Lingnan Modern Agriculture, Guangzhou 510642, China

**Keywords:** pigeon, intestine, oxidative stress, apoptosis, autophagy

## Abstract

**Simple Summary:**

Oxidative stress is a detrimental physiological state, which can adversely affect both the health and productivity of poultry, therefore strategies to identify its underlying regulatory mechanisms are crucial for poultry science. However, mechanisms contributing to oxidative stress in pigeons have not been investigated in detail previously. Therefore, we established a squab intestinal oxidative stress model, observed the changes in intestinal morphology, and detected the changes in the content of oxidative stress markers and the relative RNA expression of related genes to explore the possible mechanism of oxidative stress in the intestine of squab. The results showed that intestinal oxidative stress could lead to weight loss, intestinal morphology changes, autophagy, and apoptosis in the squabs intestine.

**Abstract:**

The purpose of this study was to evaluate the potential effect of oxidative stress on the intestine of squabs, and to explore the molecular mechanisms. A total of 360 1-day-old squabs were divided evenly into five different groups (n = 72/group): control, negative control, low, medium, and high dose groups. On the 3rd, 5th, and 7th days, squabs in the control group were not effectively treated and the negative control group were intraperitoneally injected with normal saline, whereas the H_2_O_2_ group was injected with H_2_O_2_ of 2.0, 2.5, and 3.0 mmol/kg BW respectively. On the 21st day, the serum and duodenum were collected for further analysis. The results indicated that, compared with the control group, H_2_O_2_ caused squabs weight loss and intestinal morphology damage, and these effects were enhanced with an increase in dose. Further examination revealed that the contents of oxidative stress markers in both the serum and duodenum of the H_2_O_2_ group were significantly enhanced as the dose was increased. In addition, H_2_O_2_ exposure also resulted in the lower mRNA expression of *Occludin*, *ZO-1*, *Beclin1*, *Atg5*, and *Caspase-3*, but the expression of *Claudin2* and *Bcl-2* was decreased in comparison to the control group. These findings suggested that duodenal oxidative damage was accompanied by weight loss, changes in intestinal morphology, redox status imbalance, apoptosis as well as autophagy of intestinal cells, with, effects of 3.0 mmol/kg BW of H_2_O_2_ being the most severe.

## 1. Introduction

The domestic pigeon (*Columba livia*), which is a representative of altricial birds, before 28 days old, can only be fed by the breeding pigeons. Pigeon farming, which is conducted for its excellent taste and abundant nutrition, has progressively become the fourth-largest poultry sector in China [1]. Moreover, compared with other poultry industries, pigeon meat farming is characterized by a short feeding period, where the squab growth rate is 4.32 times higher than broilers and 2.0 times higher than quails [2]. Generally, pigeons can be sold whenever their weight reaches 500 g, which generally occurs between 21 and 25 days after the hatching [3]. Therefore, strategies to attain market weight as soon as possible can improve the economic benefits of pigeon meat farming. In addition to dietary nutrition, intestine, which is the body’s primary organ for facilitating the digestion and absorption of nutrients, also has a direct impact on the growth of the pigeons. For instance, a prior study has shown that the development of the intestine of a squab lasts for 8–14 days after the shelling, but for other poultry, it is completed within 72 h after the shelling [4]. Therefore, 1~14 days after the hatching is the rapid growth period of squabs, especially the first seven days [1]. Interestingly, Gao et al. also found that the growth rate of the squab was closely related to the ability of the intestine to absorb nutrients [5]. Thus, intestinal health is a crucial factor for maintaining efficient squab production.

It has been established that under the stress of feed change, extreme environments, vaccine injection, and other factors, the body will inevitably experience oxidative stress, which can effectively accelerate the production of active substances and reactive oxygen species (ROS), thus resulting in poorer growth rates and lower-quality meat in the poultry [6]. Antioxidant defense mechanisms can protect the body against cellular abnormalities by maintaining a proper redox balance [7]. However, when the antioxidant defense system is unable to function properly and remove excessive ROS, it can lead to the oxidation of the various biological macromolecules, thus affecting their normal biological functions, such as changing the permeability of cell membranes, causing the accumulation of oxidation products and reducing their antioxidant capacity [8]. In addition, due to the unique vascular anatomical structure and convective oxygen exchange mechanism of the intestine, it has been found to be more prone to be adversely affected by oxidative stress damage [9], thereby, resulting in intestinal dysplasia and reduced antioxidant capacity [10]. Therefore, it is vital to study the molecular mechanism of intestinal oxidative stress injury and identify the oxidative stress markers to prevent and treat intestinal health problems commonly found in livestock and poultry.

Hydrogen peroxide (H_2_O_2_), representative of endogenous ROS, can be produced by almost all oxidative stress stimuli and oxidize various types of macromolecules: carbohydrates, nucleic acids, lipids, and proteins, thus, affecting the redox balance [6]. Therefore, the method of establishing an oxidative stress model by directly increasing the ROS level of the body through H_2_O_2_ has been widely recognized [8]. Referring to the previous experimental methods, we intraperitoneally injected hydrogen peroxide(H_2_O_2_) into squabs and accurately measured changes in the body weight, intestinal morphology, and oxidation marker content to reflect the potential effects of oxidative stress on the growth and development of squabs. In addition, the expression levels of the different genes involved in the intestinal barrier, apoptosis, and autophagy were assessed to investigate the possible mechanisms and relationships between these molecular indices. The findings of this study establish a solid foundation for understanding the mechanisms regulating the process of oxidative stress in the squab intestine.

## 2. Materials and Methods

### 2.1. Preparation of H_2_O_2_ Solution

Based on the average weight of squabs, a proper amount of 3% H_2_O_2_ solution (Sigma-Aldrich, St-Louis, MO, USA) with normal saline (0.75%) was mixed to prepare 2.0, 2.5, and 3.0 mmol/kg H_2_O_2_ solution till to the final volume of 1mL. The H_2_O_2_ solution was kept in a dark and dry place.

### 2.2. Experimental Design, Animal, and Management

The experimental design was approved by the Animal Care Committee of Zhongkai agricultural engineering college (Ethical approval number: 20210726). Pigeons, feeds, and sites in the experiment were provided by Meizhou Golden Green Modern Agricultural Development Co., Ltd. (Meizhou, China). A total of 360 1-day-old squabs with similar weights (16 ± 3 g) and 90 pairs of breeding pigeons that laid eggs on the same day were selected and randomly allocated into five distinct groups, each with six replicates and 12 squabs per replicate. The breeding pigeons fed the squabs with the mode of “2 + 4” and the experiment lasted for 21 d. The squabs in the control group were left untreated. On the 3rd, 5th, and 7th days of the experiment, the negative control group was intraperitoneally injected with 1ml normal saline per squab, and the H_2_O_2_ groups were injected with the same volume of 2.0, 2.5, and 3.0 mmol/kg BW H_2_O_2_ solutions per squab.

According to the nutritional needs and feeding habits of pigeons, the basic diet of the experiment consisted of pellets and raw grains in a ratio of 3:7. The pellets used in the experiment were prepared according to the nutritional requirements of poultry in National Research Council (NRC 1994). The composition of the pellets and raw grain and their nutritional levels are shown in Table 1 and Table 2, respectively. All the pigeons were provided with freewheeling access to feed and water. The environmental conditions of each group remained consistent and they were exposed every day to 16 h light and 8 h dark duration throughout the total experimental period.

### 2.3. Sample Collection

On the 21st day of the experiment, six squabs with a body weight close to the average body weight were selected from each group for collecting the sample after being subjected to fasting for 12 h. After collecting blood from the wing vein, the squabs were euthanized through the cervical dislocation and the duodenum tissues were immediately collected. The serum samples were isolated from whole blood after the centrifugation at 3000 rpm for 10 min at room temperature and frozen at −80 °C for redox state analysis. The tissue samples for morphology observation were preserved in 4% paraformaldehyde and 2.5% glutaraldehyde solution, respectively, and the other remaining samples were placed in liquid nitrogen and stored at −80 °C for qRT-PCR analysis.

### 2.4. Growth Performance

On the 7th, 14th, and 21st day, the feed consumption and the overnight (12 h) fasting body weight of squabs and breeding pigeons in each nest were recorded, respectively. Calculate the nest feed/gain ratio at the end of the experiment.

### 2.5. Hematoxylin-Eosin (HE) Staining

The duodenal samples fixed in paraformaldehyde solution (4.0%) were dehydrated with ethanol and then embedded in paraffin for continuous sectioning. The obtained sections were stained with hematoxylin-eosin and evaluated under the optical microscope. Thereafter, the intestinal wall thickness was measured by Mingmei software (Mingmei Photoelectric Technology Co., Ltd., Guangzhou, China).

### 2.6. Transmission Electron Microscopy (TEM)

The duodenal samples fixed in 2.5% glutaraldehyde were washed repeatedly with a PBS solution and then fixed again with 1% osmium tetroxide for more than 2 h, followed by staining with acid vinegar uranium dioxide. Thereafter, step-by-step dehydration was carried out in the gradient ethanol. The dehydrated samples were embedded with acetone. Then, by using the ultrathin slicing mechanism (Leica Microsystems Co., Ltd., Wetzlar, Germany), 70–100 nm ultrathin slices were prepared and installed on a 3 mm copper mesh. Finally, the slices were dyed again in the acid acetate uranium dioxide, and lead citrate solution to ensure the picture quality. Finally, the ultrastructure of the cells were observed under a transmission electron microscope (Thermo Fisher Scientific Co., Ltd., Wilmington, MA, USA).

### 2.7. Detection of Oxidative Stress Markers in Serum and Duodenum

The levels of oxidative stress in both the serum and duodenum were determined by specific markers including reactive oxygen species (ROS), malondialdehyde (MDA), protein carbonyls (PCO), and 8-oxo-deoxyguanosine (8-OHdG) using commercial pigeon Elisa kits (Meimian Co., Ltd., Yancheng, China) with a multifunctional microplate reader (Thermo Fisher Scientific Co., Ltd., Wilmington, MA, USA).

### 2.8. RNA Isolation and Quantitative Real-Time PCR Analysis (qPCR)

Total RNA was isolated from the duodenum by using a trizol reagent (Takara Biotechnology Co., Ltd., Dalian, China), and the purity, as well as concentration of RNA, was determined by Microspectrophotometry (Thermo Fisher Scientific Co., Ltd., Wilmington, MA, USA). Thereafter, by using prime script RT Master Mix Kit (Takara Biotechnology Co., Ltd., Dalian, China) RNA was reverse transcribed into cDNA. The expression of the various genes in the duodenum was determined via qRT-PCR in a fluorescent quantitative PCR instrument (Applied Biosystems Co., Ltd., California, CA, USA). The PCR reaction solution had a total volume of 20 μL and consisted of the following components according to the manufacturer’s instructions: 10 μL of SYBR Green master mix, 7 μL of ddH_2_O, 1 μL of cDNA, upstream primer, and downstream primers, respectively. PCR program consisted of one cycle at 95 °C for 2 min, 40 cycles at 95 °C for 15 s, and 60 °C for 1 min. According to the reference [11] the relative mRNA expression was calculated by employing the formula RQ = 2^−∆∆CT^. *Occludin*, *ZO-1*, *Claudin2*, and *Claudin3* genes were analyzed for intestinal barrier; *Caspase-3* and *Bcl-2* genes were analyzed for apoptosis; *Beclin1* and *Atg5* genes were analyzed for autophagy. The primer sequences of the housekeeping gene β-actin and the target genes have been listed in Table 3.

## 3. Results

### 3.1. Growth Performance

**Figure 1:**
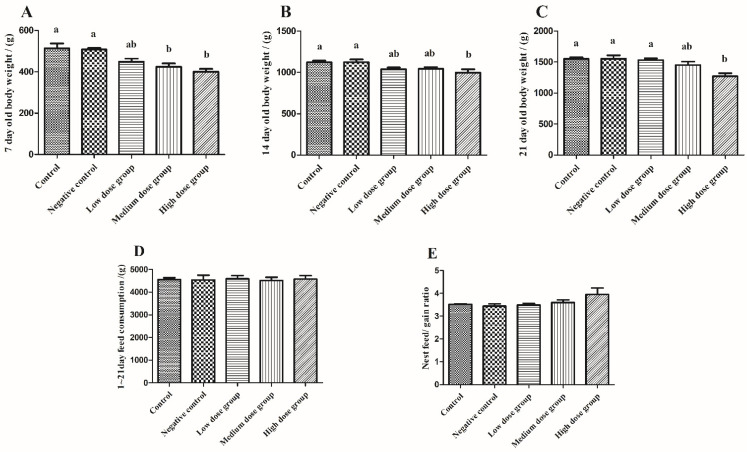
Potential effects of H_2_O_2_ injection on the growth performance. (**A**–**C**) were the 7th, 14th, and 21st day-old body weight, separately. (**D**) is 1~21 day consumption. (**E**) is nest feed/gain ratio. The control group was not subjected to any injection. The negative control group was intraperitoneally injected with 1 ml of normal saline per squab. The low-dose group, medium-dose group, and high-dose group were intraperitoneally injected with 2.0, 2.5, and 3.0 mmol/kg BW H_2_O_2_ or per squab. The data has been shown as Means ± SEM; The bars with the different lowercase letters are significantly different (*p* < 0.05). The bars with the same or no lowercase letters are no different (*p* > 0.05). n = 6.

Body weight is considered a vital indicator for measuring the economic value of meat poultry. A higher body weight is conducive to maximizing profits during production [1]. However, due to the special biological characteristics of pigeons, we have utilized the nest weight to represent the squabs body weight. As shown in Figure 1, there was no significant difference in body weight found between the control group and the negative control group (*p* > 0.05); however, intraperitoneal injection with the different concentrations of H_2_O_2_ substantially decreased the squabs body weight in varying degrees. The 7-day-old body weight of the medium and high dose groups were significantly reduced by 17.38% and 22.04%, respectively, as compared to the control group. However, the body weight of 14- and 21-day-old was also observed to be reduced to the lowest point in the high-dose group (*p* < 0.05).

### 3.2. Intestinal Histomorphology under Optical Microscope

**Figure 2:**
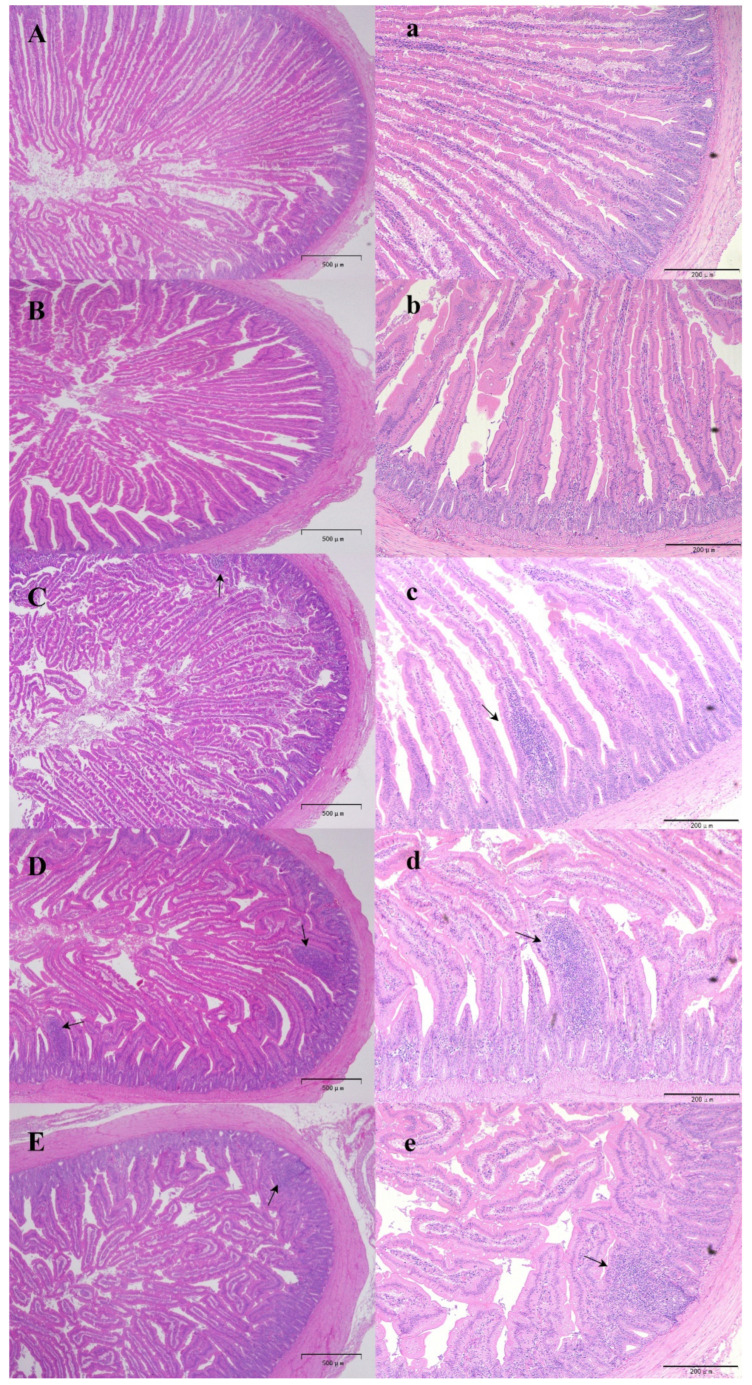
The effects of H_2_O_2_ injection on the morphology of duodenum (**A**–**E**,**a**–**e**) magnification was 40× and 100×, respectively. (**A**,**a**) show the duodenum of the control group; (**B**,**b**) show the duodenum of the negative control group; (**C**,**c**) depict the duodenum of the low-dose group; (**D**,**d**) show the duodenum of the medium-dose group; (**E**,**e**) displays the duodenum of the high-dose group; (→) Inflammatory cell infiltration.

**Figure 3:**
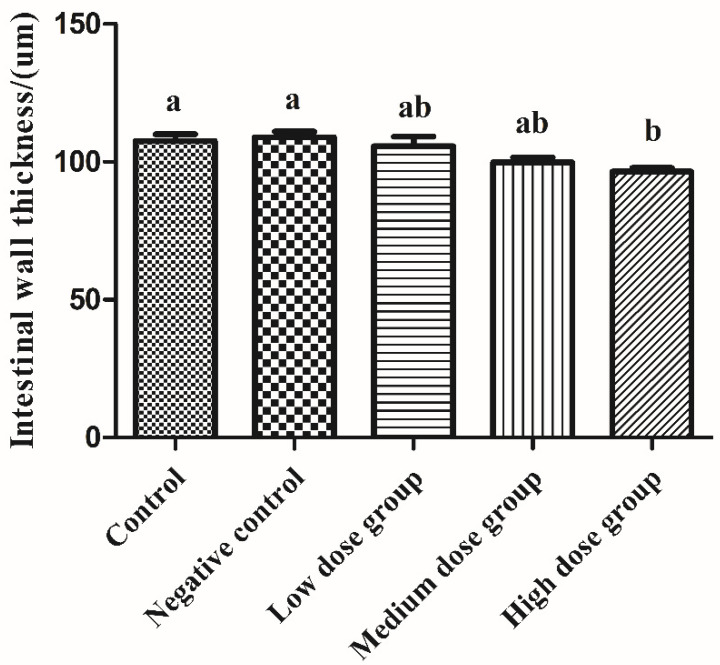
The effects of H_2_O_2_ injection on the thickness of the intestinal wall. The control group was not subjected to any injection. The negative control group was intraperitoneally injected with 1 ml of normal saline per squab. The low-dose group, medium-dose group, and high-dose group were intraperitoneally injected with 2.0, 2.5, and 3.0 mmol/kg BW H_2_O_2_ per squab. The data has been shown as Means ± SEM; The bars with the different lowercase letters are significantly different (*p* < 0.05). The bars with the same or no lowercase letters are no different (*p* > 0.05). n = 6.

When we were collecting the different samples, we found that there was a significant amount of bleeding in each segment of the intestine in the H_2_O_2_ injection group, with the duodenum being the most severe. As a result, we proceeded to investigate the specific changes in the duodenum further. We found that in general, the intestinal villi in the control group were slender and complete in shape, with properly arranged crypts. In addition, the intestinal development of the control group and the negative control group was similar (Figure 2A,B,a,b). Interestingly, H_2_O_2_ exposure caused atrophy and irregular development of the duodenal villi, hyperplasia and irregular arrangement of crypts, lymphocyte infiltration, with the highest dose exhibiting the most serious effects (Figure 2C–E,c–e). The damage to intestinal villi and crypts could significantly affect the nutrient absorption and digestion function of the squabs. As compared with the control group, the intestinal wall thickness of the high-dose was significantly thinner (*p* < 0.05, Figure 3). Thus, it was concluded that H_2_O_2_ might cause extensive damage to intestinal morphology and increase permeability.

### 3.3. Ultrastructure of Intestinal Epithelial Cells under Transmission Electron Microscope

**Figure 4:**
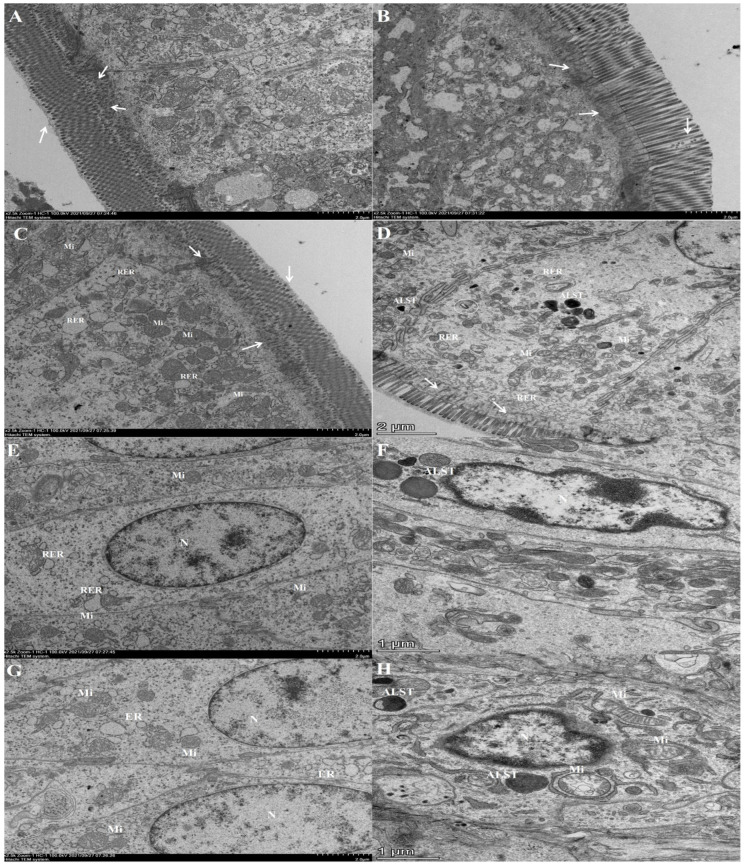
The effects of H_2_O_2_ injection on the ultrastructure of intestinal epithelial cells of the squabs. (**A**,**C**,**E**,**G**) show the intestinal epithelial cells in the control group. (**B**,**D**,**F**,**H**) depict the intestinal epithelial cells in the high-dose group. The lower end of the microfilament could connect with the terminal reticulum (→). Mitochondria (Mi). Rough endoplasmic reticulum (RER). Medullary structure (◯). Nucleus (N). Autophagic lysosome (ALST).

We found that H_2_O_2_ can cause considerable damage to intestinal villi, so we further observed the effects of H_2_O_2_ on the ultrastructure of intestinal epithelial cells. It was observed that in the control group, the duodenum was rich in mucus, the microvilli were complete and regularly arranged (Figure 4A), and the lower end of the microfilaments was intricately connected with the terminal network (Figure 4A,C). The nucleus was oval, and the nuclear chromatin was evenly distributed in the nucleus, there were euchromatin and heterochromatin in the nuclear chromatin, and no edge aggregation of the nuclear chromatin was found (Figure 4E). In addition, the cytoplasm was rich in organelles, with a large number of mitochondria, oval in shape, scattered, regular, and complete in shape, with clear internal and external membrane structures. The mitochondrial ridge structure was also clear and arranged in a tabular manner. There were many ribosomes detected on the rough endoplasmic reticulum (Figure 4A,G).

On the contrary, in the high-dose group, the mucus cells and some microvilli in the duodenum were found to be broken and shed. The lower end of the microfilament was sparsely connected with the terminal network (Figure 4B,D). The nucleus changes from a smooth oval to a deformed nucleus with an uneven surface, the heterochromatin was rich and the chromatin was condensed at the edge, which was the early manifestation of apoptosis (Figure 4F). The organelles in the cytoplasm were relatively sparse (Figure 4D). Most of the mitochondrial structures were incomplete, the double membrane structures were fuzzy, and the mitochondrial cristae structure disappears. In addition, quite a few mitochondria were surrounded by an isolation membrane, which represents the initial stage of autophagosome formation (Figure 4H). Moreover, the number of myeloid bodies, originating from the cell membrane fragments, and autophagic lysosomes increased substantially, thus indicating intense autophagy (Figure 4D,F,H). Furthermore, the ribosomes present on the rough endoplasmic reticulum fell off, the reticular cisterns were expanded, and vacuolization was also caused by endoplasmic reticulum stress (Figure 4B).

### 3.4. Content of Oxidative Stress Markers in the Serum and Duodenum

**Figure 5:**
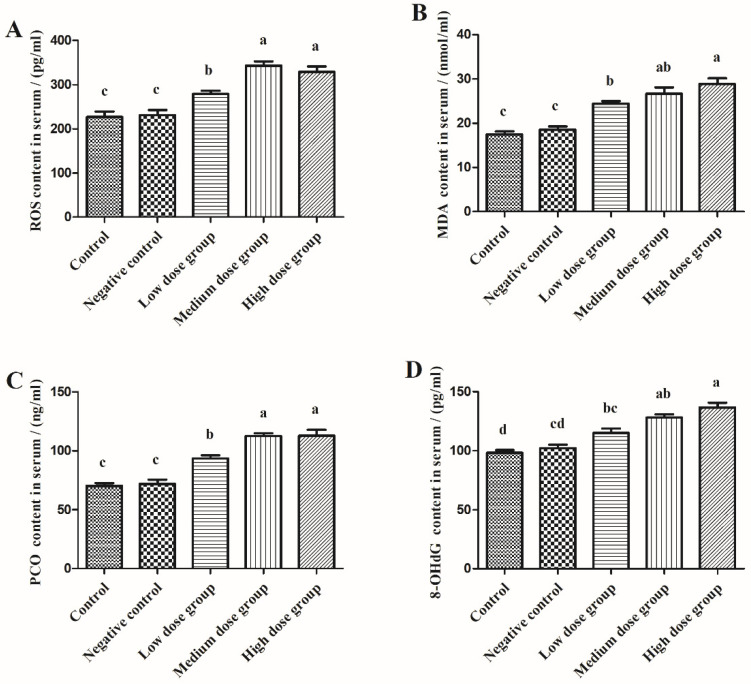
The effects of H_2_O_2_ injection on the content of oxidative stress markers in the serum of squabs. (**A**–**D**) were the ROS, MDA, PCO, and 8-OHdG content in serum, separately. The control group was not subjected to any injection. The negative control group was intraperitoneally injected with 1 ml of normal saline per squab. The low-dose group, medium-dose group, and high-dose group were intraperitoneally injected with 2.0, 2.5, and 3.0 mmol/kg BW H_2_O_2_ per squab. The data has been shown as Means ± SEM; The bars with the different lowercase letters are significantly different (*p* < 0.05). The bars with the same or no lowercase letters are no different (*p* > 0.05). n = 6.

In order to clarify the redox state of the squabs, we detected the content of oxidative stress markers in both the serum and duodenum. As shown in Figure 5, as compared with the control group, the injection of normal saline had minimal effect on the content of ROS, MDA, PCO, and 8-OHdG in serum (*p* > 0.05), but levels of these markers were significantly increased upon injection with the different doses of H_2_O_2_ (*p* < 0.05). It reflected that the redox balance was disturbed in the body by the injection of H_2_O_2_, but normal saline have no effect. Furthermore, it was observed that with an increased dose of H_2_O_2_, the contents of oxidative stress markers were increased linearly.

**Figure 6:**
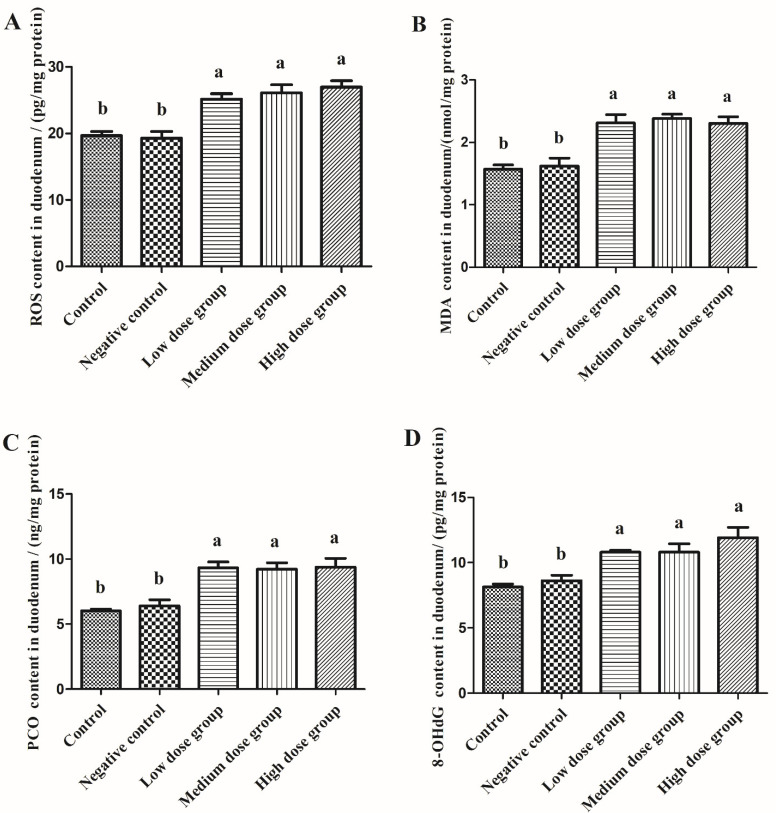
The effects of H_2_O_2_ injection on the content of oxidative stress markers in the duodenum of the squabs. (**A**–**D**) were the ROS, MDA, PCO, and 8-OHdG content in the duodenum, separately. The control group was not subjected to any injection. The negative control group was intraperitoneally injected with 1 ml of normal saline per squab. The low-dose group, medium-dose group, and high-dose group were intraperitoneally injected with 2.0, 2.5, and 3.0 mmol/kg BW H_2_O_2_ per squab. The data has been shown as Means ± SEM; The bars with the different lowercase letters are significantly different (*p* < 0.05). The bars with the same or no lowercase letters are no different (*p* > 0.05). n = 6.

As shown in Figure 6, similar to the serum, the injection of normal saline had no effect on the content of ROS, MDA, PCO, and 8-OHdG in the duodenum (*p* > 0.05), but all of these oxidative stress markers after injection of H_2_O_2_ were significantly increased (*p* < 0.05) in a dose-dependent manner.

### 3.5. Analyses of Expression of Genes Involved in the Regulation of Intestinal Barrier, Autophagy, and Apoptosis

**Figure 7:**
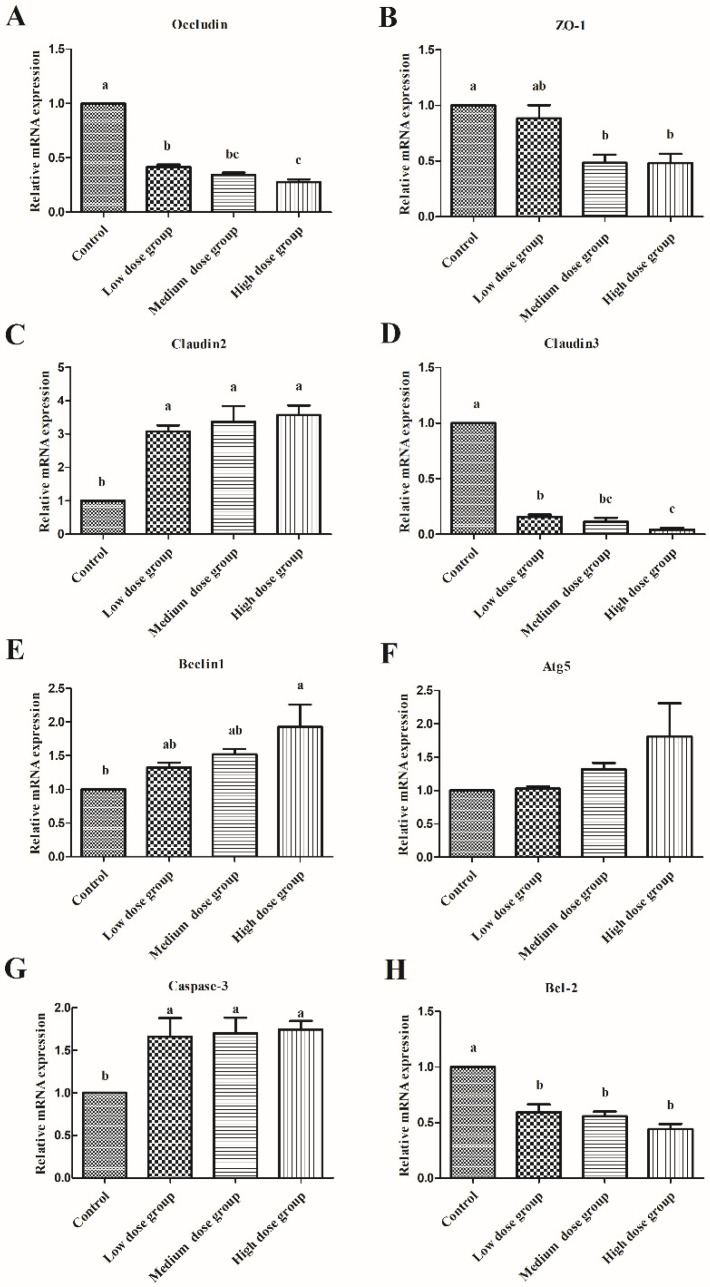
The effects of H_2_O_2_ injection on the expression of genes involved in the regulation of intestinal barrier, autophagy, and apoptosis. (**A**–**H**) were the relative mRNA expression of Occludin, *ZO-1*, *Claudin2*, *Claudin3*, *Belin1*, *Atg5*, *Caspase-3*, and *Bcl-2* in duodenum, separately. The control group was not subjected to any injection. The negative control group was intraperitoneally injected with 1 ml of normal saline per squab. The low-dose group, medium-dose group, and high-dose group were intraperitoneally injected with 2.0, 2.5, and 3.0 mmol/kg BW H_2_O_2_ per squab. The data has been shown as Means ± SEM; The bars with the different lowercase letters are significantly different (*p* < 0.05). The bars with the same or no lowercase letters are no different (*p* > 0.05). n = 6.

We further detected the expression of various genes involved in the intestinal barrier (*Occludin*, *ZO-1*, *Claudin2*, and *Claudin3*), autophagy (*Belin1* and *Atg5*), and apoptosis (*Caspase-3* and *Bcl-2*). As shown in Figure 7, it was found that in comparison with the control group, H_2_O_2_ injection in the squabs increased the mRNA expression of *Claudin2* and *Caspase-3* in the duodenum (*p* < 0.05), but significantly decreased the expression of *Claudin3*, *occludin*, and *Bcl-2* expression (*p* < 0.05). In addition, further comparison showed that injection of high dose H_2_O_2_ could significantly increase the expression of *Belin1* (*p* < 0.05), and significantly decrease the expression of *ZO-1* (*p* < 0.05).

## 4. Discussion

A broad variety of oxygen-containing molecules, which possess significant oxidation potential are collectively referred to as reactive oxygen species (ROS). It has been established that the imbalance between ROS production and scavenging is the main source of oxidative stress, which can arise from the impairment of the ability of the antioxidant defense to remove excessive ROS from the body [12]. For instance, in Fenton’s reaction and Fe^2+^ ions can react to produce highly reactive OH- which can accelerate the generation of ROS, and constitutes the main mechanism underlying oxidative damage [13]. The design of cell redox models frequently uses hydrogen peroxide (H_2_O_2_), a potent oxidant solvent. Similarly, intraperitoneal injection of H_2_O_2_ can also lead to rapid production of ROS in chickens which can cause extensive oxidative tissue damage [6]. Since squab intestine is not fully developed and the ability to stimulate anti-stress actions is relatively weak, it is more prone to suffer intestinal oxidative stress [14]. This can result in a number of major complications, including decreased growth performance, reduced feed intake, and poorer meat quality, all of which inevitably can result in greater economic losses. We also observed in our investigation that an intraperitoneal injection of 3.0 mmol/kg H_2_O_2_ caused considerable weight loss in the squabs. When under oxidative stress, the intestine is the primary organ that experiences the earliest attack, is most vulnerable, and is also the slowest to recover [15]. Thus, we detected the content of ROS in both the serum and duodenum after exposure to H_2_O_2_. We found a considerable increase in the content of ROS in the serum and duodenum, which corroborated the findings of Chen et al. [8]. It has been reported that an overdose of ROS can cause the rapid oxidation of the polyunsaturated fatty acids that constitute the cell membrane, improper protein folding, altered enzyme activity, and DNA strand fragmentation [16]. These alterations can significantly compromise the structural and functional integrity of the cell membrane, thereby impairing the ability of the tissues to conduct their biological functions [17]. The oxidative damage indicators MDA, PCO, and 8-OHDG can be used to assess the degree of oxidative damage in the various macromolecules such as lipids, proteins, and DNA [16]. As there was no significant difference observed between the control group and the intraperitoneal injection of normal saline in the squabs in the current study, the effect of normal saline on the experiment was ruled out. However, after intraperitoneal injection of H_2_O_2_, the content of oxidative stress markers in the duodenum of squabs increased significantly, thus, suggesting that the oxidative stress model was successfully established. In addition, consistent with the previous observations, 2.96 mmol/kg BW H_2_O_2_ injection was found to elevate MDA, PCO, and 8-OHDG levels in the chicken liver [18]. Absorption and digestion are considered the most basic functions of the intestine, and the duodenum plays a key role in the process of digestion. When intestinal oxidative stress occurs, digestion, absorption, and barrier functions are usually disturbed. Therefore, the oxidative stress in the intestine that was generated by the injection of medium- and high-doses of H_2_O_2_ could be the probable reason contributing to the weight loss in the squabs.

The intestine is not only an important organ for the absorption and digestion of nutrients but also acts as a pivotal barrier for the body to effectively resist the complex external environment and maintain the stability of the internal environment [19]. It has been reported that the complete intestinal barrier plays a critical role in preventing intestinal bacteria, and toxic or allergic substances from entering the human body through the intestinal tract [20]. Moreover, the complete intestinal villi and crypt serve as an important line of defense against unfavorable digestive problems [21]. Because the physical integrity of the barrier is primarily regulated by the balance between the proliferation and death of stem cells in the crypt, the deeper the crypt, the slower the stem cells might renew [22]. Intestinal villi are protrusions on the intestinal mucosa, which are mainly composed of intestinal stem cells that are undergoing constantly differentiation and proliferation. The villi can increase the surface area of the intestinal wall to markedly improve the efficiency of nutrient absorption [4]. However, when certain bacteria and their toxic products can stimulate intestinal epithelial cells, their proliferation and shedding could be accelerated, which might damage the integrity of the intestinal mucosal barrier [23]. The thickness of the intestinal wall is closely related to nutrient absorption and intestinal permeability. It has been reported that oxidative stress leads to edema and congestion of intestinal mucosa, lodging, rupture, and necrotic shedding of villi in the broilers [24]. Similarly, we found upon analysis of the duodenal section that H_2_O_2_ can cause intestinal villus destruction, cause the crypt to become deeper, and lead to arrangement abnormality, intestinal wall thinning, as well as inflammatory cell infiltration. Further examination of the intestinal ultrastructure revealed that H_2_O_2_ injection could result in significant thinning of the mucous layer, destruction of the microvilli structure, and an increase in permeability, which was consistent with the HE staining results of intestinal morphology.

Based on the above findings, we further detected the expression level of tight junction-related proteins at the molecular level. Occludins are the major family of tight junction structural proteins that were first discovered. Claudins is a transmembrane protein that can be stably expressed in most epithelial cells, acting as the main adhesion molecule between cells [25]. They can connect with the cytoskeleton by binding ZOs functional proteins, interact with proteins of the neighboring cells through the cell membrane, and create a stable structure that can prevent the invasion of foreign materials [26]. In addition, any specific protein with abnormal structure and function can lead to the weakening of the tight junction structure, lead to an increase of intestinal mucosal barrier permeability, and cause diarrhea-type irritable bowel syndrome (IBS), and other related symptoms. Moreover, a number of previous studies have demonstrated that oxidative stress can substantially decrease the gene expression of the different tight junction proteins [27]. In this study, oxidative stress significantly reduced the mRNA expression of *Ocludin*, *Claudin-3*, and *ZO-1* in the H_2_O_2_ injection group, whereas, expression of *Claudin2* related to cellular membrane permeability was significantly increased, thus indicating that oxidative stress destroyed the integrity of the tight connection between the duodenal cells in the squabs, thereby causing intestinal mucosal barrier dysfunction. An increase in intestinal permeability and a decrease in mRNA expression of immunoglobulins and tight junction protein has been reported when squabs are under early weaning stress [28]. To sum up, these findings indicate that H_2_O_2_ can destroy the integrity of intestinal morphology and reduce the mRNA expression of various tight junction proteins through stimulating oxidative stress, thereby increasing intestinal permeability and affecting the normal function of the intestine.

During animal breeding, the continuous attack of feed-derived toxins, warm environment, harmful bacteria, as well as adverse growth conditions, and other stimulating factors can lead to a rapid rise in the content of ROS, disrupt the balance of the intestinal redox system, induce cell apoptosis, hinder the orderly updating of crypt chorionic axis, and damage the health of the body [29]. Thus, analysis of the duodenum’s ultrastructure revealed that H_2_O_2_ caused swelling of the endoplasmic reticulum, mitochondrial rupture, swelling or vacuolization, cell nuclear deformation, and chromatin aggregation at the edge of the nucleus, thus indicating that intestinal cells were in initial stages of apoptosis. Apoptosis is a process of programmed cell death used to maintain proper physiological functions and balance the metabolism, which is essential for maintaining homeostasis [30]. However, if apoptosis in healthy cells is aberrantly enhanced, it can interfere with cellular metabolism and damage the structural integrity [31]. Furthermore, prior studies have suggested that the excessive production of ROS can lead to intestinal cell apoptosis, thus increasing intestinal permeability, promoting the migration of harmful substances, and leading to a series of intestinal and systemic inflammatory reactions [14]. When apoptosis occurs, decreased expression of *Bcl-2* can promote the Cyt-C release, and released Cyt-C can then activate caspase proteins. Caspase-3 plays a vital role in the execution of apoptosis [32,33]. It was found that compared with the control group, the mRNA expression of *Caspase-3* in the H_2_O_2_ group increased, but that of *Bcl-2* decreased. Based on the above analysis, H_2_O_2_ can decrease the mRNA expression level of *Bcl-2*, thereby activating the caspase-3 protein and inducing apoptosis. In addition, our results were consistent with the previous studies, which indicated that enhanced oxidative stress in the intestinal tract of mice could lead to the increase of Bax/Bcl-2 ratio, stimulate the release of Cyt-C and promote the activation of Caspase-9/3 pathway leading to apoptosis [34].

In addition, during oxidative stress generated by excessive accumulation of ROS, a greater number of the damaged mitochondria and autophagosomes were observed in the cells of the high-dose group. In order to protect themselves from oxidative damage, the cells have evolved a sophisticated self-protection mechanism. Damaged organelles could be isolated in autophagosomes and then degraded by the lysosomes, which can contribute to the maintenance of intestinal homeostasis and significantly reduce further oxidative damage [35]. The formation of autophagosomes depends on the participation of autophagic protein Atgs [36]. Until now, 37 kinds of Atg proteins have been discovered in mammalian cells, with Atg5 being a major promoter of the autophagy process and can play a critical role in the production of autophagosomes [37]. Beclin1 is an autophagy regulatory protein, which can mediate the localization of other autophagic proteins in phagocytes and thus regulate autophagosome production and maturation [38]. Thus, enhanced expression of both these markers in cells suggests that the autophagy level has been increased. In our study, the expression of autophagic proteins *Beclin1* increased significantly, which was consistent with the results of a large number of autophagosomes observed in TEM. Autophagy and apoptosis are two major types of programmed cell death, with a difference in terms of inducement, signal pathway involvement, and mechanism. Interestingly, in this study, compared with the control group, there was no substantial difference observed in the expression of autophagy-related genes in the low-dose group and medium-dose group, whereas apoptosis was activated. At the same time, the expression of various genes regulating apoptosis and autophagy was significantly increased in the high-dose group. These findings indicated that there could be a potential relationship between autophagy and apoptosis, which might be related to the degree of oxidative stress. However, additional experiments are required to fully understand the detailed mechanism and crosstalk between these two cell death pathways.

## 5. Conclusions

In conclusion, the results of this study show that intraperitoneal injection of H_2_O_2_ can effectively induce the excessive production of oxidative markers, which will have a negative impact on the redox status of squabs. The molecular mechanism of oxidative damage may be related to the oxidation of different biomacromolecules, the destruction of the intestinal structure, and the induction of abnormal autophagy and apoptosis of intestinal epithelial cells. Under the experimental conditions, the oxidative stress model of the pigeon intestine can be established by injecting 3.0 mmol/kg BW H_2_O_2_.

## Figures and Tables

**Table 1:** The composition and nutrient levels of the pellets in a basic diet (air-dry basis).

Item	Content	Nutrient Levels ^2^	
Ingredients (%)		ME/(MJ/kg)	11.90
Corn	40.00	CP(%)	22.97
Wheat bran	4.18	EE(%)	4.64
Wheat middling	10.00	CF(%)	5.73
Soybean meal	32.50	Ca(%)	1.09
Rice bran meal	5.00	TP(%)	0.59
Soybean oil	4.00	Lys(%)	1.26
Premix ^1^	0.55	Met(%)	0.36
CaHPO_4_	0.59		
Limestone	1.73		
Lys	0.12		
Met	0.12		
NaHCO_3_	0.08		
Choline chloride	0.08		
Antifungal agent	0.10		
NaCl	0.20		
Zeolite	0.75		
Total	100.00		

^1^ The Premix provided per kilogram of the diet: Vitamin A 7, 350 IU, Vitamin D_3_ 1, 650 IU, Vitamin E 11.25 mg, Vitamin K 1.95 mg, Vitamin B_1_ 1.50 mg, Vitamin B_2_ 4.80 mg, Vitamin B_6_ 3.00 mg, Vitamin B_12_ 0.015 mg, Nicotinic acid 30.00 mg, Folic acid 0.75 mg, D-Pantothenic acid 9.00 mg, Biotin 0.03 mg, Cu (as copper sulfate) 3.00 mg, Fe (as ferrous sulfate) 105.00 mg, Mn (as manganese sulfate) 90.00 mg, Zn (as zinc sulfate) 60.00 mg, I (as calcium iodide) 0.30 mg, and Se (as sodium selenite) 0.20 mg. ^2^ Among the nutrients, metabolizable energy (ME) was calculated value, whereas other nutrient levels indicators were measured values.

**Table 2:** Composition and nutrient levels of the raw grain in the basic diet (air-dry basis).

Item	Content	Nutrient Levels ^1^	
Corn	37.50	ME/(MJ/kg)	13.00
Sorghum	31.25	CP	12.06
Wheat	15.625	EE	3.21
Peas	15.625	CF	3.08
		Ca	0.03
		TP	0.3
		Lys	0.47
Total	100	Met	0.15

^1^ Among the nutrients, metabolizable energy (ME) was calculated value, whereas other nutrient levels indicators were measured values.

**Table 3:** The primer sequences used for the Real-Time PCR.

Genes	Forward Primer Sequence (5′→3′)	Reverse Primer Sequence (5′→3′)	Product Size (bp)	Accession Number
*β-actin*	F: CCCATCTACGAAGGCTACGC	R: CTTGATGTCACGCACAATTTC	149	XM_005504502.2
*Occludin*	F: CAGGACGTGGCAGAGGA	R: GTGGAAGAGCTTGTTGCGT	105	XM_005509325.2
*ZO-1*	F: GAACCAAAGCCAGTGTATG	R: GGTCCCCTTCCTCTAATC	161	XM_021299314.1
*Claudin2*	F: GTGCAGATGGGAACAAGGT	R: GAGCCAAGGAAGCTACGG	119	XM_021283269.1
*Claudin3*	F: ACCTCATCCCCGTCTCCT	R: CAGCCCACGTAGAGCGA	109	XM_005515008.2
*Beclin1*	F: AGCTGGAGGACGTTGAGAAA	R: AGCTCCAGTTGCTGTCTCTT	139	XM_021280982.1
*Atg5*	F: GTCCAAGGTTTGTGGCTGTT	R: CAGAATGGGAACAGCACTGG	188	XM_005509471.2
*Caspase-3*	F: CCTACCTGCCAGCAAGTCTA	R: CTTGCAGCATCTGCATCTGT	159	XM_005509733.3
*Bcl-2*	F: TACCTCCGAGACCCTGAGAA	R: CAGCAACAGGGAGAGAGGAA	161	XM_005500178.2

## Data Availability

The datasets of the current study are available from the corresponding author upon reasonable request.
